# Disruption of chromosome 11 in canine fibrosarcomas highlights an unusual variability of *CDKN2B *in dogs

**DOI:** 10.1186/1746-6148-5-27

**Published:** 2009-07-31

**Authors:** Jesús Aguirre-Hernández, Bruce S Milne, Chris Queen, Patricia CM O'Brien, Tess Hoather, Sean Haugland, Malcolm A Ferguson-Smith, Jane M Dobson, David R Sargan

**Affiliations:** 1 Veterinary Medicine, University of Cambridge, Madingley Road, Cambridge, UK

## Abstract

**Background:**

In dogs in the western world neoplasia constitutes the most frequently diagnosed cause of death. Although there appear to be similarities between canine and human cancers, rather little is known about the cytogenetic and molecular alterations in canine tumours. Different dog breeds are susceptible to different types of cancer, but the genetic basis of the great majority of these predispositions has yet to be discovered. In some retriever breeds there is a high incidence of soft tissue sarcomas and we have previously reported alterations of chromosomes 11 and 30 in two poorly differentiated fibrosarcomas. Here we extend our observations and present a case report on detail rearrangements on chromosome 11 as well as genetic variations in a tumour suppressor gene in normal dogs.

**Results:**

BAC hybridisations on metaphases of two fibrosarcomas showed complex rearrangements on chromosome 11, and loss of parts of this chromosome. Microsatellite markers on a paired tumour and blood DNA pointed to loss of heterozygosity on chromosome 11 in the *CDKN2B*-*CDKN2A *tumour suppressor gene cluster region. PCR and sequencing revealed the homozygous loss of coding sequences for these genes, except for exon 1β of *CDKN2A*, which codes for the N-terminus of p14^ARF^. For *CDKN2B *exon 1, two alleles were observed in DNA from blood; one of them identical to the sequence in the dog reference genome and containing 4 copies of a 12 bp repeat found only in the canine gene amongst all species so far sequenced; the other allele was shorter due to a missing copy of the repeat. Sequencing of this exon in 141 dogs from 18 different breeds revealed a polymorphic region involving a GGC triplet repeat and a GGGGACGGCGGC repeat. Seven alleles were recorded and sixteen of the eighteen breeds showed heterozygosity.

**Conclusion:**

Complex chromosome rearrangements were observed on chromosome 11 in two Labrador retriever fibrosarcomas. The chromosome alterations were reflected in the loss of sequences corresponding to two tumour suppressor genes involved in cell-cycle progression. Sequencing of *CDKN2B *across many different breeds revealed a widespread polymorphism within the first exon of the gene, immediately before the ankyrin coding sequences.

## Background

Neoplasia is profoundly important as a cause of morbidity and mortality in the domestic dog, *Canis lupus familiaris*. In addition to its importance as a working animal and a pet species, the dog is an important intermediate model species for human tumour biology. It has a relatively large body size, often displays responses to cytotoxic or other therapeutic agents comparable to humans, and has a relatively high natural incidence of several cancers with similar biology to human tumours. Dogs have been used as models for cancer therapy, such as in osteosarcoma, in the giant dog breeds, oral melanoma and non-Hodgkins lymphoma, amongst others (reviewed in [1,2]. In general, the same tumour types are recognised in humans and dogs; for instance, the classification of soft tissue sarcomas of dogs largely follows the human system [3]. Studies of cytogenetic and genetic changes in canine soft tissue sarcomas may reveal more about their aetiology.

In a previous study, primary cell cultures were obtained from two poorly differentiated fibrosarcomas [4]. Both tumours (which were name-coded LE and ME) came from adult female Labrador retrievers. Chromosome painting revealed they both had abnormalities in chromosomes 11 (CFA11) and 30 (CFA30). One tumour (LE) contained four translocation chromosomes involving CFA4, CFA11, CFA27 and CFA30 (tumour karyotype 2n = 78; t(4;11;30), t(11;27); t(27;11); t(30;4); der 11 (del 11q)); the second one (ME) had a deletion of CFA11q and trisomy of CFA30 (tumour karyotype 2n = 79; der11; +30). An attempt to isolate the translocation chromosomes by Fluorescence Activated Chromosome Sorting had not allowed complete purification of these chromosomes but PCR of sequence tagged sites (STS), on sorted preparations, had shown that sequences in the first 7 Mb of CFA 27 and the last 20 Mb of CFA11 were present in the LE tumour genome, but missing when t(27;11) was excluded. Rearrangement in the Transforming Growth Factor Beta Receptor 1 gene (*TGFBR1*) was implied as only a portion of the exonic sequences could be obtained by PCR of DNA in this preparation. In addition to this altered copy of *TGFBR1*, the tumour still had at least one normal coding sequence of the gene. This tumour sequence was identical to that obtained from blood DNA of the same individual. At least one normal coding sequence of *TGFBR1 *was also present in the tumour ME. Here we extend our search for molecular alterations of CFA11 that could be linked to canine sarcomas and present a case report involving the loss of heterozygosity in a region harbouring two tumour suppressor genes.

## Results

### Further characterisation of derivative chromosomes by BAC hybridisation

**Figure 1:**
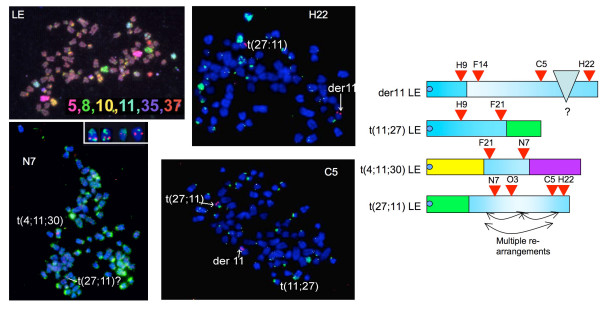
**Chromosome painting of metaphases spreads from canine fibrosarcoma LE**. Chromosome painting of tumour LE (6 colour paints, colours as shown) shows 4 derivative chromosomes containing CFA11 DNA. To look at the distribution of CFA11 BACs, metaphases were hybridised with a mixed paint of CFA 4, 27 and 30 (green) and a single BAC paint as indicated in examples shown, (red). For N7, where the t(27;11) is difficult to identify because of a high green background, additional hybridising chromosomes from other metaphases are shown; inset at twice the scale. Diagrams summarise hybridisation of all BACs with each derivative containing chromosome 11. The red arrowheads denote BACs consistently binding to the chromosomes shown. Chromosomes in the diagram are CFA11 in blue (mid blue -centromeric; light blue -telomeric); CFA27 green; CFA4 yellow; CFA30 purple.

To obtain a more detailed description of rearrangements in chromosome 11 in these fibrosarcomas, eleven canine BACs spaced at intervals along chromosome 11 were used to probe tumour metaphases, in some cases in combination with chromosome paints identifying translocation partners (Table 1). For LE, the distribution of BAC hybridisation suggests that the der11 and t(4;11;30) chromosomes originate from one chromosome 11 whilst t(11;27) and t(27;11) originate from the other (Figure 1). However, the pattern of rearrangements in derivative chromosomes is complex. The der11 chromosome contains both centromeric and telomeric sequences in an inverted arrangement, whilst central parts of this chromosome 11 are found in t(4;11;30) (BACs between 375-F21 and 381-N7 locate here). t(11;27) contains the centromere proximal region of CFA11. Although no BACs more telomeric than 375-F21 hybridised to this chromosome, chromosome painting suggests that more than half of the normal CFA11 is present, so there may be some further interstitial insertion of sequences not represented by the BACs used here. More telomeric BACs hybridised to t(27;11) but, again, these showed rearrangement such that 381-H22 has a sub-telomeric position whilst BACs which lie distal to this in the normal chromosome are now placed near the centre of the derivative. The translocation break/fusion point is close to RP81_381-N7 with BACs derived from centromere proximal sequence found distal to this position in the fusion chromosome.

**Table 1:** BACs used for in situ hybridisation.

BAC name	CFA	Genomic position (bp)	Signal on LE der11	Signal on LE t(4;11;30)	Signal on LE t(11;27)	Signal on LE t(27;11)	Signal on ME der11
386-H9	11	6153594	+		+		+

385-C1	11	22151575	NA*	NA	NA		+

375-F21	11	24354423		+	+		+

372-O3	11	32432415	+			+	+

372-K9	11	33384887	NA	NA	NA		+

381-N7	11	41509254		+		+	+

376-M15	11	46649071	NA	NA	NA		-

381-H22	11	49355967	+			+	+

373-C5	11	59457191	+			+	-

373-M14	11	65702272	NA	NA	NA		-

381-F14	27	38704742	+			+	-

*NA = Data not available.

**Figure 2:**
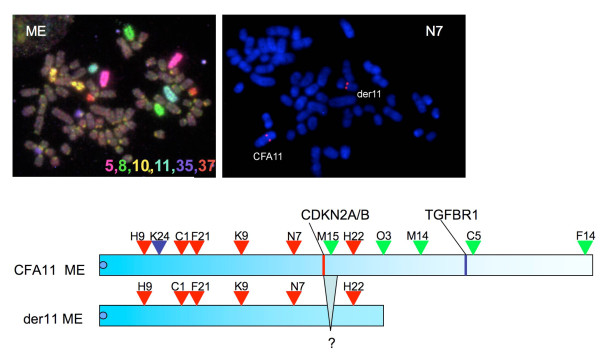
**Chromosome painting of metaphases spreads derived from fibrosarcoma ME**. Chromosome painting of tumour ME (6 colour paints, colours as shown) shows a der11 with ± 40% of the genomic material of the normal chromosome. An example BAC hybridisation is shown, using RP81_381-N7 (red). Diagrams of the normal and derivative chromosome show hybridisation positions of BACs: red arrowheads – BACs consistently binding to both chromosomes; green arrowheads -those not hybridising to the derivative; blue arrowhead -consistent hybridisation to the normal but inconsistent to the der 11 chromosome.

For ME, BACs telomeric of RP81_381-H22 did not hybridise to the derivative chromosome, but RP81_381-H22 itself did (Figure 2). There was also lack of signal from RP81_376-M15 on the derivative, suggesting interstitial deletion of the derivative chromosome in the area of this BAC. In independent RH mapping studies the BAC 381-F14 is placed on chromosome 11 at position 8810, telomeric to 373M14 [5].

### Loss of heterozygosity

**Figure 3:**
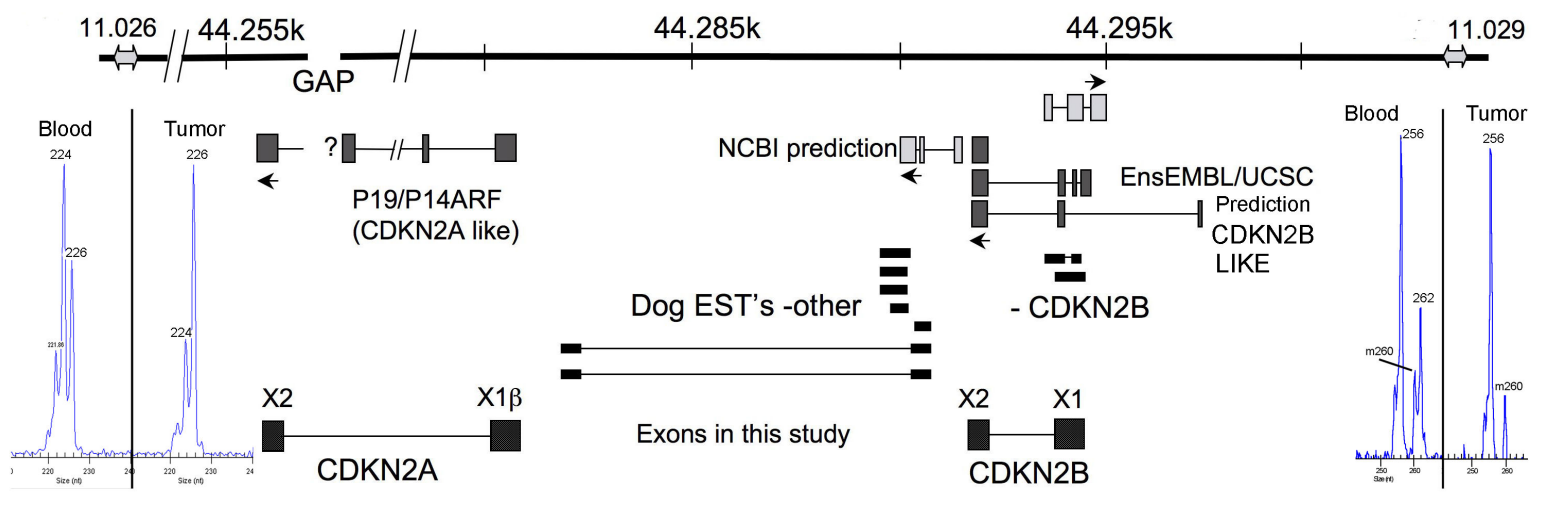
**Map of the canine region containing the *CDKN2B-CDKN2A *locus**. Top, position of CAMC11.026, CAMC11.029, and of the gap in the genomic sequence assembly. Middle, microsatellites presenting loss of heterozygosity; also, position of predicted *CDKN2 *related exons (darker grey), other unrelated exons (light grey), and canine ESTs in the database (black). Bottom, exon designations used here.

For LE, genomic DNA from blood and tumour was available, while only tumour DNA could be obtained for sample ME. Loss of heterozygosity on CFA11 was analysed for the LE fibrosarcoma. Forty-five microsatellites distributed across the chromosome were studied (Table 2). Complete loss of heterozygosity (loss of the 262 bp allele) was observed for marker CAMC11.029, and a substantial loss (of the 224 bp allele) for marker CAMC11.026 (22% remaining signal) (see Figure 3). The region between these two markers contains sequences orthologous to the human genes coding for the proteins p15^INK4B^, p16^INK4A ^and p14^ARF^. The first one is coded by the cyclin-dependent kinase inhibitor 2B gene (*CDKN2B*), while the other two are coded by cyclin-dependent kinase inhibitor 2A (*CDKN2A*) [reviewed in [6]]. A marker (CAMC11.027) within the canine *CDKN2B-CDKN2A *region was uninformative (homozygous in blood and tumour DNA for sample LE). Most other markers showed no change between tumour and blood, but one further marker, (FH4031, 29.9 Mb proximal from CAMC11.026), showed partial loss of one allele (37% remaining signal). For marker CAMC11.004 (10 Mb from CAMC11.026) a discrepancy was observed in the results corresponding to the blood and the tumour; the former presented a heterozygous genotype, while the tumour had a single allele that was different to either of those observed in the blood.

**Table 2:** CFA11 loss of heterozygosity.

Feature*	Sequence type	Mb Distance From Top	LE blood genotype§	LE tumour genotype	Observations
CAMC11.009	Microsatellite	4.985028	387, 387	387, 387	

AHT137	Microsatellite	5.829562	148, 148	148, 148	

REN161P13	Microsatellite	7.106779	189, 189	189, 189	

CAMC11.008	Microsatellite	8.593416	195, 195	195, 195	

FH3203	Microsatellite	8.623136	89, 89	89, 89	

CAMC11.010	Microsatellite	11.425726	307, 307	307, 307	

FH4031	Microsatellite	14.237434	312, 331	312, 331	LOH

REN286P10	Microsatellite	17.844923	182, 185	182, 185	

REN242K04	Microsatellite	19.23201	328, 328	328, 328	

CAMC11.022	Microsatellite	20.94395	164, 169	164, 169	

CAMC11.021	Microsatellite	24.758282	242, 250	242, 250	

REN142O09	Microsatellite	26.338024	256, 261	256, 261	

CAMC11.020	Microsatellite	29.356331	332, 332	332, 332	

CAMC11.001	Microsatellite	31.434645	264, 264	264, 264	

FH2004	Microsatellite	32.161602	240, 240	240, 240	

CAMC11.003	Microsatellite	33.777804	285, 285	285, 285	

FH2710	Microsatellite	34.47053	187, 187	187, 187	

CAMC11.004	Microsatellite	34.561666	247, 363	249, 249	

FH2874	Microsatellite	35.143202	177, 177	177, 177	

CAMC11.005	Microsatellite	35.801351	327, 327	327, 327	

CAMC11.006	Microsatellite	36.813802	321, 325	321, 325	

FH2319	Microsatellite	37.54361	304, 334	304, 334	

FH2982	Microsatellite	41.069406	361, 379	361, 379	

CAMC11.019	Microsatellite	41.843895	243, 243	243, 243	

FH2706	Microsatellite	43.991845	201, 201	201, 201	

CAMC11.026	Microsatellite	44.172674	224, 226	224, 226	LOH

*CDKN2A*	Gene, exon 2	44.255474	Present	Missing	

Contig_16328	contig end	44.258553			

Gap in dog sequence		44.258554			

*CDKN2A*	Gene, exon 1α	Undetermined			

Gap in dog sequence		44.260435			

Contig_16329	contig start	44.260436			

*CDKN2A*	Gene, exon 1β	44.280332	Present	Present	

CAMC11.027	Microsatellite	44.288351	306, 306	306, 306	

*CDKN2B*	Gene, exon 2	44.291018	Present	Missing	

*CDKN2B*	Gene, exon 1	44.293951	Heterozygous	Missing	

CAMC11.029	Microsatellite	44.304276	256, 262	256, 256	LOH

CAMC11.017	Microsatellite	45.277711	207, 238	207, 238	

C11.868	Microsatellite	48.547399	216, 216	216, 216	

CAMC11.016	Microsatellite	50.214468	223, 223	223, 223	

CAMC11.015	Microsatellite	52.827522	209, 213	209, 213	

CAMC11.014	Microsatellite	55.676158	268, 268	268, 268	

FH2019	Microsatellite	56.07231	211, 211	211, 211	

FH3238	Microsatellite	57.734062	276, 276	276, 276	

REN249L05	Microsatellite	58.631801	175, 175	175, 175	

*TGFBR1*	Gene	59.220624			

CAMC11.024	Microsatellite in *TGFRB1 *intron 7	59.239311	310, 310	310, 310	

CAMC11.013	Microsatellite	59.911747	338, 346	338, 346	

CAMC11.023	Microsatellite	61.260282	265, 277	265, 277	

REN147O02	Microsatellite	61.367981	243, 243	243, 243	

CAMC11.012	Microsatellite	63.816291	218, 222	218, 222	

CAMC11.011	Microsatellite	67.610944	212, 212	212, 212	

C11.873	Microsatellite	67.767854	136, 144	136, 144	

FH3065	Microsatellite	72.608147	185, 185	185, 185	

DGN13	Microsatellite	72.807881	303, 326	303, 326	

*Microsatellites are listed according to their position in Ensembl's CanFam 2.0 (July 2008). The position of exons within the *CDKN2B-CDKN2A *gene cluster is provided, along with the position of the gap between contigs 16328 and 16329, and the location of *TGFBR1*. ^§^The numbers in the genotype columns correspond to the PCR product sizes (in base pairs) of the alleles, as determined by the sequencing instrument.

### *CDKN2B *and *CDKN2A *sequencing from tumour DNA

*CDKN2A *and *CDKN2B *have not been fully described in the dog. No mRNAs or ESTs that completely define these genes are available, and gene prediction programmes do not fully agree with each other or with those EST that are available. In addition, the area is extremely GC rich and has proved difficult to clone or to sequence, containing a gap in the CamFam 2.0 genome assembly [7,8]. Alignments with human and other *CDKN2A/B *RNAs appear to be the best guide to gene structure. We used these to design PCR primers. To determine whether the expected remaining copy of these genes had any mutations in tumour LE, the predicted exonic sequences, and their flanking intronic sequences, were investigated. Since the gap in the dog reference sequence is where *CDKN2A *exon 1α is expected to be present (Table 2 and Figure 3), it was not studied. However, a PCR product could be obtained for the predicted exon 1β; this sequence was identical to that observed in blood DNA from the same individual, and to the sequence in the reference genome. In contrast to this, for *CDKN2A *no PCR products of exon 2 could be obtained from tumour LE's DNA in spite of repeated attempts, but the sequence was obtained from blood DNA and it contained no difference when compared to the reference genome.

**Figure 4:**
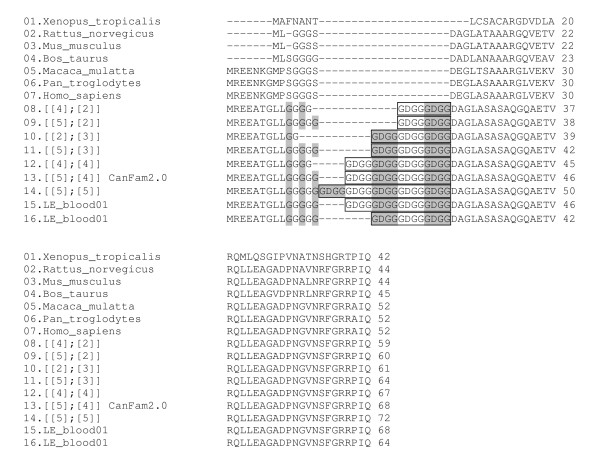
**Predicted canine *CDKN2B *exon 1 amino acid sequences**. Alternate Gly residues in the polymorphic region have a grey background, while the GlyAspGlyGly repeats are enclosed in a frame and alternate repeats also have a grey background. In LE's tumour DNA the exon was missing, but not in peripheral blood where it was heterozygous. Alleles are identified in a simplified way (e.g., [2][3]] corresponds to p. [Gly10[2];Gly15_Gly18[3]]).

When using DNA from tumour as template, no PCR products could be obtained for *CDKN2B *exons 1 and 2 and their flanking intronic sequences. In LE's DNA from blood, the sequence of *CDKN2B *exon 2 was the same as in the reference genome. Exon 1 of this same gene could not be PCR-amplified from tumour LE but it was amplified using DNA from blood, and then cloned and sequenced. The results showed that this individual was heterozygous, having one allele with the same sequence as the reference genome (g. [109GGC[5]; 124GGGGACGCCGCC[4]]) as well as a shorter allele (g. [109GGC[5]; 124GGGGACGCCGCC[3]]). At the amino acid level the first allele would have five Gly residues followed by four tandem copies of the sequence GlyAspGlyGly (p. [Gly10[5]; Gly15_Gly18[4]]), while the second allele would consist of five Gly followed by only 3 copies of the tetrapeptide (p. [Gly10[5]; Gly15_Gly18[3]]) (Figure 4).

### *CDKN2B *exon 1 polymorphism in the dog

**Figure 5:**
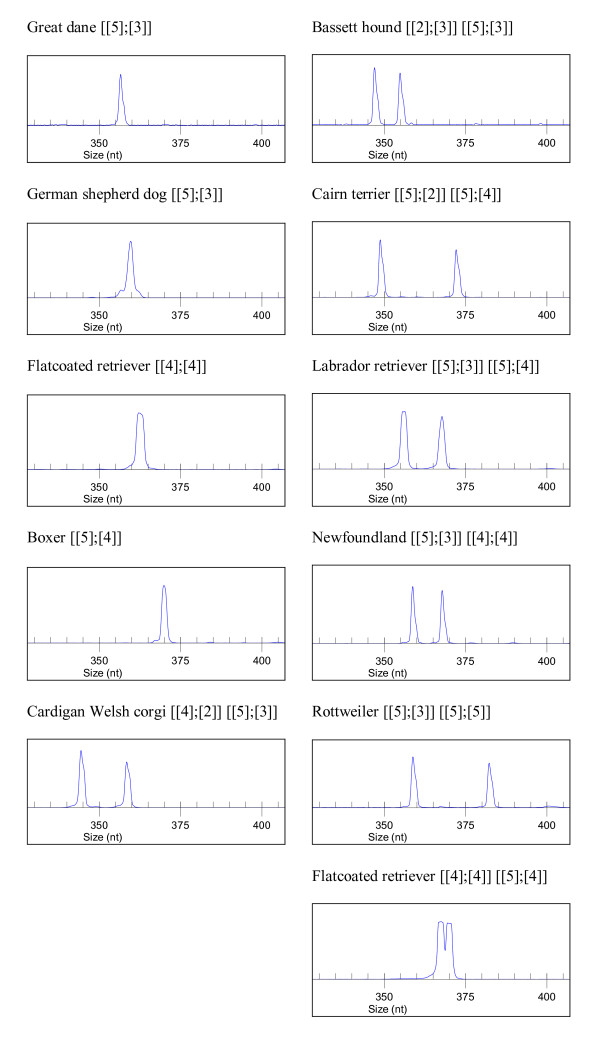
***CDKN2B *exon 1 genotype determination in dogs of different breeds**. Dye-labelled PCR products were separated by capillary electrophoresis. Fragments representing all different sizes were sequenced to confirm the identity of the alleles.

**Figure 6:**
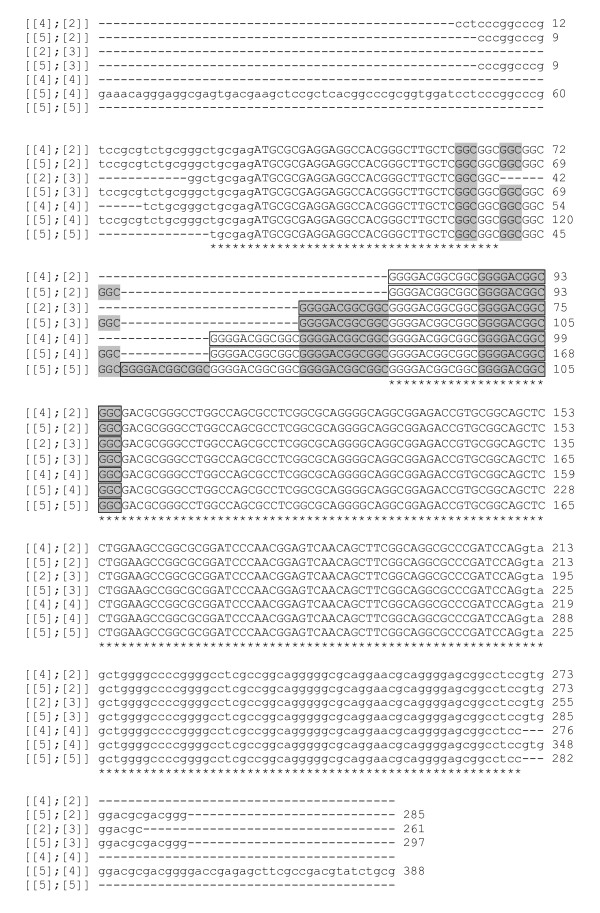
***CDKN2B *exon 1 alleles observed in canine blood and tumour samples**. Alternate GGC triplets coding for Gly have a grey background. The dodecameric nucleotide sequence (GGGGACGGCGGC) coding for the GlyAspGlyGly repeats are within a box, with alternate repeats having a grey background. Intronic sequences are in lowercase. Alleles are identified with a short version of their description; e.g., [4][2]] stands for g. [109GGC[4];124GGGGACGGCGGC[2]]. The [5][4]] allele corresponds to the reference genome (CanFam2.0).

To investigate whether the shorter allele could be related to the development of the neoplasia, the exon was was amplified using DNA from blood of 141 dogs of 18 different breeds. The size of the PCR products was determined by capillary electrophoresis and products corresponding to the different sizes were sequenced directly (Figure 5). Seven different alleles were found (see Figure 6, and Additional file 1: Distribution of *CDKN2B *exon 1 alleles in various dog breeds). The most frequent allele was p. [Gly10[5]; Gly15_Gly18[4]], which corresponds to the reference sequence and was found in all breeds studied. Alleles p. [Gly10[5]; Gly15_Gly18[3]] and p. [Gly10[4]; Gly15_Gly18[4]] were also observed. Additionally, 4 alleles were rarer in this sample set; the shortest being p. [Gly10[4]; Gly15_Gly18[2]] in Cardigan Welsh corgis, and the longest p. [Gly10[5]; Gly15_Gly18[5]] in a Rottweiler. Among these blood samples, 24 corresponded to flat-coated retrievers; of these 11 had histiocytic sarcoma and 13 were non-affected dogs aged 10 years or more (See Additional file 1: Distribution of *CDKN2B *exon 1 alleles in various dog breeds). The distribution of the alleles was not significantly different between these two sets (chi-square test p = 0.55). All breeds except boxer (15 individuals examined) and Shih Tzu (two individuals only) were polymorphic at this position

### Other *CDKN2B *and *CDKN2A *polymorphisms in the dog

To search for additional polymorphisms in these two genes, exon 2 of *CDKN2B *was studied using DNA from blood samples from a Bernese mountain dog, four boxers, nine flat-coated retrievers (four with histiocytic sarcoma and five free from neoplasia), two golden retrievers and a Labrador retriever. No polymorphisms were seen in the coding region or the flanking intronic bases.

**Figure 7:**
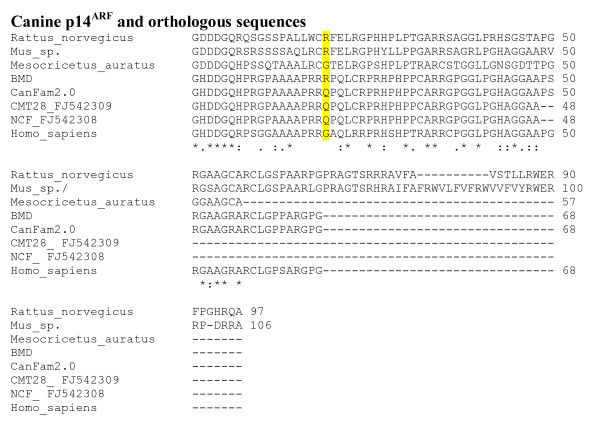
**Polymorphism in exon 2 of canine *CDKN2A *in the p14^ARF ^reading frame and alignment to orthologous sequences**. The dog polymorphic residue is highlighted in yellow.

**Figure 8:**
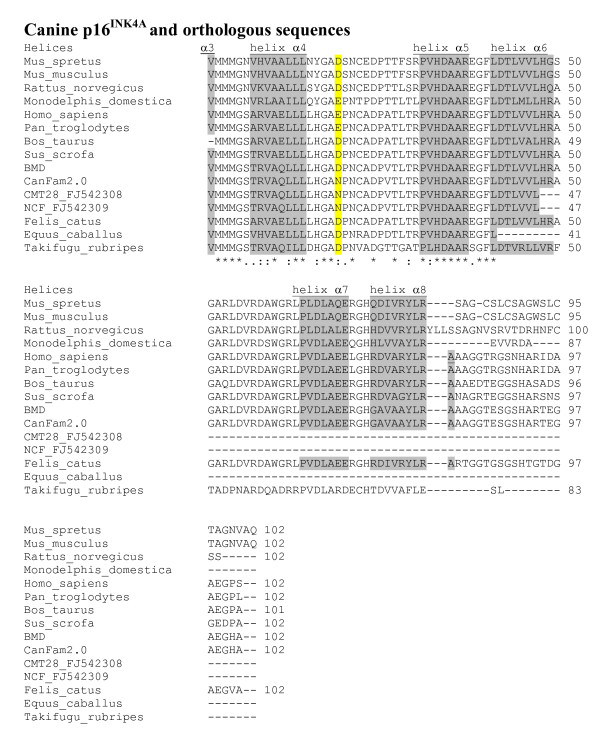
**Polymorphism in exon 2 of canine *CDKN2A *in the p16^INK4A ^reading frame and alignment to orthologous sequences**. The canine polymorphic position is highlighted in yellow in the loop joining the second and third ankyrin repeats. The α helix residues of the ankyrin repeats have a grey background. CanFam2.0 -dog reference genome; BMD -Bernese Mountain dog with the alternative Asp residue; CMT28_FJ542308 -canine mammary tumour cell line; NCF_FJ542309 -cultured canine fibroblasts.

Exon 2 of *CDKN2A *was also sequenced from DNA obtained from blood samples from three Bernese mountain dogs, four flat-coated retrievers with histiocytic sarcoma, and five flat-coated retrievers free from tumours, two golden retrievers and four Labrador retrievers. A coding SNP was identified that led to a non-synonymous change in the predicted codon 19 of exon 2 that is shared by both p16^INK4A ^and p14^ARF^. The polymorphism was a g.1206A>G transversion that corresponds to a p.Gln79Arg change in the predicted amino acid sequence of p14^ARF ^(Figure 7) and an Asn to Asp change in the p16^INK4A ^reading frame (Figure 8). Both alleles were found in all breeds studied, except in the boxer where all four individuals were homozygous for the reference sequence. In the flat-coated retrievers half of the histiocytic sarcoma cases were GG homozygous and the rest AG heterozygous, while two controls were homozygous GG and three were heterozygous.

## Discussion

A previous analysis of two canine fibrosarcomas with rearrangement of chromosome 11 was followed up by BAC mapping. In both tumours this revealed that rearrangements were more complex than had been seen using chromosome painting. In LE all chromosomes containing fragments of chromosome 11, except t(11;27), showed interstitial deletions and or rearrangements. In ME a deletion around BAC M15 was observed. Loss of heterozygosity (LOH) on chromosome 11 was studied in LE's tumour. Marker CAMC011.004 (34.56 Mb) presented an allele in the tumour which was different from any of those in the blood. This result was confirmed in two independent experiments. This marker is within a gene desert, with no proven transcripts encoded or genes modelled within about a megabase. Our data does not show whether this marker is close to a rearrangement. FH4031 (14.24 Mb), which shows LOH, is also some way from the nearest known gene. In this case more proximal markers are not informative. It seems likely that, in the tumour, the der11 and t(4;11;30) chromosomes have lost some sequences in this region, which is near the proximal chromosome breakpoint in both.

Loss of heterozygosity was also observed in two markers which bracket a region encompassing the *CDKN2B*-*CDKN2A *gene cluster. Even though the microsatellite study pointed to loss of heterozygosity rather than complete loss of the region containing *CDKN2A *and *CDKN2B*, no PCR products could be obtained from the tumour for the genes themselves, except for *CDKN2A *exon 1β. The sequence of this exon showed no differences with the reference one. A microsatellite within the gene cluster could be amplified but it was homozygous in blood and thus non-informative regarding loss of heterozygosity. This microsatellite is 8 kb away from *CDKN2A *exon 1β and 3 kb from *CDKN2B *exon 2, which could not be detected in the tumour DNA. These results suggest that two copies of parts of chromosome 11 are present in the tumour, while other regions are present only once, and still others, close to the latter, are completely missing. This complex pattern is reminiscent of the alterations observed for t(27;11), where *TFGBR1 *exons 3 to 6 and 9 were observed, but not the rest of the exons [4]. This agrees also with the recent observation on the complexity of chromosome rearrangements in human tumours [9].

Both approaches, BAC mapping and loss of heterozygosity, point to alterations on canine chromosome 11, in the region equivalent to human chromosome 9p21 which is commonly deleted in human tumours [10] and contains the genes *CDKN2B *and *CDKN2A*. The first codes for the protein p15^INK4B^, while the second codes for p16^INK4A ^and p14^ARF ^which are proteins derived from alternate exon 1 sequences and use different reading frames for the common exon 2 [11,12]. p16^INK4A ^and p14^ARF ^regulate the retinoblastoma protein 1 (pp105^RB1^) and the tumour protein 53 (TP53), respectively. These form part of the two pathways most commonly disrupted in human tumours. Recently a new protein, smARF, also coded by *CDKN2A*, was identified in mouse and human and localised in the mitochondria [13]. It has been shown to inhibit cell growth and proliferation, and to induce apoptosis in a TP53-independent manner [14,15]. *CDKN2A *also codes for the proteins p12 and p16γ which are less well understood [16,17]. In mice, disruption of either p19^Arf ^(the orthologue of human p14^ARF^) or p16^Ink4b^, or both, results in increased predisposition to tumour development [18-21]. In humans, homozygous deletions are the most frequent type of mutation involving these genes [22], as opposed to the combination of mono-allelic deletion followed by the mutation of the remaining copy of the gene, which is the common pattern in other tumour suppressor genes [23]. However, alterations affecting only one of p15^INK4B^, p16^INK4A ^or p14^ARF ^have been reported [11]. Apart from homozygous deletions, 5'CpG methylation has also been observed in some tumours [12,24-26]. In human patients with soft tissue sarcomas, loss of *CDKN2B *and *CDKN2A *is associated with reduced survival [27], while in dogs, absence or reduced levels of p16^INK4A ^have been reported in melanoma tumours and cell lines [28], as well as in osteosarcoma cell lines [29]. In canine non-Hodgkin's lymphoma (NHL), deletion of p16^INK4A ^or loss of CFA11 have been observed in high-grade T-cell NHL, without comparable alterations seen in high-grade B-cell NHL or in low grade tumours [30]. In this same paper, p16^INK4A ^methylation was observed in a single low-grade T-cell lymphoma. In addition to this, in two canine mammary tumour cell lines, CMT12 and CMT27, no expression of p16^INK4A ^was observed, while in a third cell line (CMT28) increased expression was detected [31]. Upon transfection of a complete human p16^INK4A ^cDNA, these cell lines lost most of the characteristics of the transformed phenotype [31]. The sequences of CMT28 and of canine fibroblast cultured cells were reported to differ from the reference sequence, although the nucleotide and the aminoacid sequences are identical to the canine reference sequences used here for the second exon of p16^INK4A ^and p14^ARF ^(Figure 7 and Figure 8).

*CDKN2A *exon 1β codes for the N-terminus portion of p14^ARF. ^The first amino-acid residues of this protein are relatively well conserved and have an important role in binding MDM2 while the rest of the protein, containing a nucleolar localization signal, is poorly conserved and may be dispensable [11,32,33]. In chicken, exon 1α of *CDKN2A *has been lost and no p16^INK4A ^protein is produced [34]. Exon 1β is spliced to the still existent exon 2 but the predicted protein terminates at the end of exon 1β, with no residues from exon 2. In spite of this, the protein is able to bind MDM2 thus preventing the induction of TP53 degradation and the inhibition of *TP53 *expression [34]. It is therefore likely that exon 1β codes for the amino acid residues required for p14^ARF ^to perform its normal role. Moreover, aberrant p16^INK4A ^transcripts have been observed in tumours [35] and some of them are translated and are able to function in cell-cycle control [36]. In the present study only exon 1β of *CDKN2A *was observed in LE's fibrosarcoma. It is possible that an abnormal version of p14^ARF ^could have been produced; it is unknown whether the remaining exon 1β would have been spliced to another sequence, generating an abnormal transcript and, possibly, a shorter version of p14^ARF^, as is in the chicken. In the current study, a polymorphism was observed in exon 2 causing missense mutations in both p16^INK4A ^and p14^ARF^. The polymorphic residue is in the loop joining the second and thitd ankyrin repeats of p16^INK4A^. This residue is on the surface of the protein [37]. Although it does not interact with CDK6, it does seem to have a role in stabilising the structure of p16^INK4A ^[38,39]. In other species this position is occupied by the acidic residues Asp or Glu, so the polar neutral Asn residue seems to be unique to dogs. However, other structurally related proteins with ankyrin repeats have Asn in the equivalent position (e.g., p18) [37].

Polymorphisms in short repeat sequences within coding exons have been observed before [40,41], whilst somatic repeat instabilities are well documented in other species both in tumours and in some degenerative diseases. The putative first exon of *CDKN2B *in dogs has a predicted GlyAspGlyGly repeat that is not found in other species. This exon is represented in the database by two ESTs [GenBank: CX985268 and GenBank: DN749168]. The multiplicity of alleles in this gene may have resulted from occasional misalignments during crossover events between homologous chromosomes, leading to the expansion and contraction in the number of GGC triplet and GGGGACGGCGGC dodecamer repeats. No instances were observed of nonsense mutations in this region, nor were indels found that would produce shifts in the reading frame. In the case of flat-coated retrievers, which show a high predisposition to developing histiocytic sarcomas, cases and controls were found to have a similar distribution of exon 1 alleles. Moreover, the sequences obtained from genetic material from blood samples of various breeds suggest that the variation in the number of Gly residues and in the number of GlyAspGlyGly repeats is a normal polymorphism in this species.

p15^INK4B^, coded by *CDKN2B*, and the rest of the members of the inhibitor of kinase 4 (INK4) protein family (p16^INK4A^, p18^INK4C ^and p19^INK4D^) have ankyrin repeats that play an important role in the folding of the protein and in intermolecular interactions with other proteins, such as the cyclin-dependent kinases [39]. p15^INK4B ^and p16^INK4A ^are similar proteins that appear to be the result of a gene duplication event [42]; both have 4 ankyrin repeats and they appear to function in a similar manner and may be interchangeable [43]. However, p16^INK4A ^has been studied in more detail. It is known, for example, that this protein's loops 1 and 2, as well as some of the ankyrin repeats themselves, interact with CDK6 [38,44-46]. The GlyAspGlyGly repeats predicted to exist in canine p15^INK4B ^are immediately before helix α1 of the first ankyrin repeat. Hence the polymorphism may not disrupt the folding or function of the protein which is dependent on the ankyrin repeats and the loops connecting them. These GlyAspGlyGly repeats would introduce a region of small glycine residues punctuated by charged aspartates causing a difference in molecular size at the N-terminus of the protein.

p15^INK4B ^is induced by transforming growth factor, beta 1 (TGFB1) [47-49]; this induction may be direct, through the binding of TGFB1 to a sequence within the promoter of *CDKN2B *[50]. In a previous study we showed that the breakpoint of the translocation chromosome t(27;11) involves *TGFBR1 *[4] which codes for a receptor to TFGB1. However, the tumour still contained a complete coding sequence of the gene. It seems, therefore, that the pathway is disrupted at the level of p15^INK4B ^and p16^INK4A ^through the homozygous deletion of their coding sequences, while the possibility still remains that an abnormal p14^ARF ^could be present.

## Conclusion

In fibrosarcomas studied in this work complex rearrangements of canine chromosome 11 were observed, causing changes in genes in the TGFB – p14^INK4B ^pathway. Study of *CDKN2B *showed unusual variants present in exon 1 of the gene in different breeds of dog, as a result of a simple repeat sequence. No instances of missense or frameshifting mutations were observed. However, it is possible that the genomic plasticity of *CDKN2B *observed here is connected with the rather high frequency of cancers in the domestic dog.

## Methods

### Tumours and blood samples

The two tumours, corresponding to two dogs name-coded LE and ME, have been described previously [4]. Both are fibrosarcomas from adult Labrador retriever females from the pet population. Blood samples were obtained from excess material used for the purpose of clinical diagnostic studies of dogs admitted to the Queens Veterinary School Hospital of the University of Cambridge.

### Fluorescence-in-situ Hybridisation

Chromosome paints for seven colour FISH and other combinations of single colour canine chromosome paints were prepared and used as described previously [51]. BACs were from the RPCI-81 canine BAC library [52], and DNA's from them were labeled with biotin-dUTP or digoxigenin-dUTP by nick translation. For hybridization, 300 ng of labeled DNA in 1 μl TE representing one chromosome paint probe mix, or 50 ng of labeled BAC DNA, was mixed with 14 μl hybridization buffer (55% formamide,11% dextran sulphate, 2.2 × SSC, 45 mM sodium phosphate pH7,1.1× Denhardt's solution (Sigma), 0.5 mM EDTA, 45 μg/ml canine Cot-1 DNA), heated at 70C for 10 minutes, then left 20–30 minutes at 37°C to allow pre-annealing of the Cot-1 DNA. Metaphase preparations on glass slides were denatured, dried and hybridized to the denatured probes. Hybridization was overnight at 37°C in a humid chamber. Slides were washed two × 5 mins in 50% formamide/50% 2 × SSC at 45C followed by two × 5 mins in 2 × SSC at 45°C and a final wash in 4 × SSC, 0.01% Tween20 before application of fluorochrome-conjugated antibodies.

Biotin labeled probes were visualized using Cy3-avidin (single colour FISH) or Cy5-avidin (multi-colour FISH) (1:500 dilution, Amersham). FITC labeled probes were visualized using sequential application and washing of rabbit anti-FITC (1:200) and FITC-labeled goat anti-rabbit antibodies (1:100). After 20 minutes incubation at 37°C, excess antibody was washed off by three 5 minute washes in 4 × T at 42°C. Slides were mounted with Vectashield containing DAPI and images were captured as before [53]. Fluorescence signals were captured separately as 8-bit black and white images through appropriate excitation filters, normalized, and merged into a 24-bit colour image. 10 – 15 metaphases were captured for each probe mix.

### DNA extraction form blood

For one of the patients (LE) blood was available and DNA was extracted using the QIAamp DNA Blood Mini kit (QIAGEN) according to the manufacturer's instructions. This same extraction procedure was used for the bloods used to study the *CDKN2B *exon 1 polymorphism in 18 breeds.

### Loss of heterozygosity analysis

For individual LE, for which tumour and blood DNA was available, loss of heterozygosity analysis was performed. Microsatellite markers were chosen along the canine chromosome 11; the location of non CAMC markers and their primer sequences appear in the dog reference genome in Ensembl [7]. To fill the gaps between adjacent markers, and to increase the density in some regions of interest, such as those around the *CDKN2B*-*CDKN2A *locus and the *TFGBR1 *gene, additional microsatellites were identified from the dog reference sequence: appropriate contig sequences were analysed with Tandem Repeat Finder version 4.00 [54] and primers were designed manually or using Primer3 [55,56]. These markers carry the prefix CAMC in the name. All PCR reactions had a final volume of 10 μl, with 1 to 10 ng of genomic DNA as template, 5 U of Taq polymerase (Invitrogen), a final concentration of 0.2 pmol/μl for each primer, 1.5 mM of MgCl_2 _and 0.25 mM of each dNTP. For markers with the CAMC prefix, the PCR products were labelled indirectly using a two-step amplification procedure. For these markers, the forward marker-specific primer had a T7 universal sequence (5'-TAATACGACTCACTATAGGG-3') added to its 5' end. These markers, with forward and reverse primer sequences (omitting the T7 sequence from the forward primer), are: CAMC11.001 (AAATGGTCCATCAGAG, ACTAGCTAAGAACTTCATGG); CAMC11.002 (AGTGCTTTGATGCTGA, GAAAGACCTACATTAGTACC); CAMC11.003 (ATAAGACCAAACCTAC, TCTCATGTTTCTGAAGAGTG); CAMC11.004 (AAGTAGCCATACTAAT, CTCAGACCTTATCTATTTGG); CAMC11.005 (ATGGGTATTAAAGACG, CCAAACTGAACCAACATGAG); CAMC11.006 (AACACTCAGTCAAGCC, ATCGGACTCCTTCCATGCTG); CAMC11.007 (CTTGGATAAGCTCTGC, TTCTGGACAGTACTACATTC); CAMC11.008 (GGAGGGGGATTGGTTTTGAT, ATGCTCACTCACTCGCATGT); CAMC11.009 (CCTGTTGGAACTGGAGCTTT, TTTGGGATAATCTAAAGCAAATC); CAMC11.010 (GGGTACCTTCTTTCGGCATT, CATAGCTGACTCCCTTGAAG); CAMC11.011 (CCCTCATACCACGGCTTTTA, ATGACCAAAACTGCCCAGAG); CAMC11.012 (TTCTTCTTTGGGAGTTGCACA, TTCAGCTCCCTTGGAAACTC); CAMC11.013 (ATGGTAGATGGAGGACCTGA, CAGGATGTGTGAAGGGGAG); CAMC11.014 (GGCAGAGGGTGGGTAAAATA, GCCGCCCTGTATGCCTTTT); CAMC11.015 (ACATGTGAAGGCAACCATTTG, TTGCTTAGACAGTATGAAATATG); CAMC11.016 (TTATCCCTGGAAACCCAACAT, TGGCCAAGGTTACACAGTAG); CAMC11.017 (GATGGGACAAGTGTCAAAGG, TTGTCCTCTGAGCAAGTCTG); CAMC11.018 (CAGGAGCCCACTCCTTTTTA, GAGTGACTGTGCGTGTGTG); CAMC11.019 (GCACATGAGGACCCTTCAAT, ATGTCATGGAAAACTGCAAGC); CAMC11.020 (AGTAAACACTACCACATCTGG, TCAGGTGGAGCCAGGTTTTA); CAMC11.021 (ATCATGGAACCCAGAGCAAC, AAGCCAGCTCATCAAGGAGA); CAMC11.022 (TCCTGAGAAAGGCCTGGATA, GCCCGTCAAGTTAGTGAGG); CAMC11.023 (CTGCCAACTCCTCCTCTGT, CCCTCCCAACTGTTCCAAAT); CAMC11.024 (AACAGCAGGACAAAGTCTGC, ATATCATTCGTGGCCCTTCC); CAMC11.026 (TCATGGGTCATGAGATGGAC, GCCCTCATGAATGGGATTAG); CAMC11.027 (CACAGAATAACTCAATAGGTTG, ACTTCTGTGAAGTGCCTTATG); CAMC11.029 (GGTTCAAGTTCAGAATGCTTG, GTTTAGCGTTAGCGCCTGC). For the first PCR these unlabelled primers were used; 1 μl of these products were then used as template for a second PCR reaction with a T7 primer having a D2, D3 or D4 label (Proligo) on its end. The first PCR reaction was a touch-down PCR with an initial annealing temperature of 66°C; this temperature was decreased by 1°C for 12 cycles, followed by 30 cycles with an annealing temperature of 54°C. For the second PCR 25 cycles were performed with an annealing temperature of 54°C. For all PCRs, each step had a duration of 20 sec. For markers whose name does not have the prefix CAMC, the PCR amplifications were performed with a single reaction using forward primers labelled with D2, D3 or D4 (Proligo). After PCR amplification, regardless of whether one or two step reactions had been performed, all PCR products were diluted 1:8 to 1:12 using sterile water. Two microlitres of each diluted product were mixed with 0.15 μl of D1-labelled GenomeLab DNA size standard 600 (Beckman Coulter) and 30 μl of GenomeLab Sample Loading Solution containing formamide (Beckman Coulter). The products were then run in a CEQ8000 sequencer (Beckman Coulter) and genotypes were retrieved with the instrument's Fragment Analysis software. This same program was used to determine loss of heterozygosity, by comparing the peaks obtained from the blood sample and for the matching tumour. Loss of heterozygosity was noted when either peak showed a greater than 30% loss of signal.

### *CDKN2B *and *CDKN2A *sequencing

Primers were designed for studying *CDKN2B *and *CDKN2A *exonic sequences plus the flanking intronic bases. The primer names, sequences (5' to 3'), starting and ending annealing temperatures for touchdown PCR, and PCR product sizes were as follows. For *CDKN2B *exon 1 primers 1729 (GAAACAGGGAGGCGAGTGAC) and 1730 (CGCAGATACGTCGGCGAAGC), 70°C to 58°C, 388 bp; for *CDKN2B *exon 2 primers 1731 (GAAATGGTCCACCTGTCCCTG) and 1732 (CACCGTGACTCAAGTCTCCTG), 72°C to 60°C, 470 bp; for *CDKN2A *exon 1b primers 2257 (GAGCTTCCACCCCTAGAAAC) and 2258 (CGGCTCCGAGATCGGAGG), 72°C to 60°C, 526 bp; and for *CDKN2A *exon 2 primers 1727 (CTTGTAGCGGCATCTGCATGG) and 1728 (TGCTCTGGGCTGCGGAAG), 72°C to 62°C, 459 bp. *CDKN2B *and *CDKN2A *sequences are GC-rich, so they were PCR-amplified with AccuPrime GC-Rich DNA polymerase (Invitrogen) using Buffer A for that enzyme. PCRs were done in 40 μl, using 6 to 30 ng of genomic DNA, a final concentration of 02.pmol/μl for each primer and 1.6 U of the polymerase. All amplifications were done using touchdown-PCR. PCR products, except for *CDKN2B *exon 1, were cleaned with the QIAquick PCR purification kit (QIAGEN), sequenced with the GenomeLab DTCS kit (Beckman Coulter) and ran in a CEQ8000 sequencer. Sequences were analysed and aligned against the canine genomic reference sequences using the Sequence Analysis and Sequence Investigator modules on the sequencer. Differences with respect to the reference sequence were confirmed by sequencing the complementary strand. *CDKN2B *exon 1 PCR fragments from tumour samples were cloned and then sequenced. AccuPrime's GC-rich polymerase proofreading activity yields blunt-ended products; to clone the fragments into the PCR4-TOPO vector of the TOPO TA Cloning Kit for Sequencing (Invitrogen) a 3' A-overhang was incorporated to the PCR products by incubating them with 1 μl of Taq polymerase (Invitrogen) at 72°C for 10 min. Sequencing reactions with the GenomeLab DTCS kit were performed according to the manufacturer's protocol for plasmid templates except that betaine (Sigma) was added to a final concentration of 1 M. The reference sequences used were CanFam 2.0, assembly May 2006, Genebuild Sep 2008 [7,8] for the canine genome; [EMBL: FM946072] for *CDKN2B *and [EMBL: FM883643] for *p14*^*ARF*^/*CDKN2A*. Polymorphisms and mutations in these sequences are described following the recommended nomenclature [57,58].

### *CDKN2B *exon 1 polymorphism search

To determine the *CDKN2B *exon 1 alleles present in a collection of dog DNAs of different breeds, the exon was amplified, along with the flanking intronic sequences, using a D4 (Sigma) labelled forward primer 1729 and an unlabelled reverse primer 1730. The length of the product, according to the reference sequence in CanFam2.0 is 388 bp [7]. PCRs were performed using the KAPA2G Robust PCR Kit (Kapabiosystems, Cape Town, South Africa), with buffer B and Kapa Enhancer 1, in a volume of 10 μl, with a minimum of 8 ng of template and a touchdown-PCR starting with an annealing temperature of 72°C, and decreasing by 1°C per cycle to 59°C, followed by 25 cycles with an annealing temperature of 58°C. Annealing, extension (at 72°C) and denaturation (at 95°C) were 20 sec long each. The products were diluted 1:8 with sterile water and loaded on a CEQ8000 sequencer (Beckman Coulter) to determine the size of the PCR products with the Fragment Analysis module of the instrument; 18.25% of the samples were retested to confirm the results. 22.63% of the samples, representing the different allele sizes observed, were amplified again from genomic DNA and sequenced directly with the GenomeLab DTCS kit following the procedure recommended by the manufacturer for GC-rich sequences. Cleaned products were separated on the CEQ8000 at 60°C and results analysed as in the sequences described in the previous section. Multiple alignments of nucleotide and predicted amino acid sequences were done with ClustalW2 [59] at the EMBL-EBI website [60] or using JalView 2.3 [61] and then manually edited. GenBank accession numbers for p15^INK4B ^amino acid sequences used for the alignments were [*Xenopus tropicalis *GenBank: AAH75575.1, *Rattus norvegicus *GenBank: EDL97746.1, *Mus musculus *GenBank: AAH02010.1, *Bos taurus *GenBank: NP_001069362, *Macaca mulatta *GenBank: XP_001107263.1, *Pan troglodytes *GenBank: ENSPTRP00000035618, *Homo sapiens *GenBank: P42772]. The sequences for p16^INK4A ^were [*Takifugu rubripes *GenBank: CAC12808.1, *Monodelphis domestica *GenBank: AAC23669.1, *Bos taurus *GenBank: XP_612365.1, *Equus caballus *GenBank: AAC97110.1, *Sus scrofa *GenBank: CAB65454.1, *Mus spretus *GenBank: AAD00236.1, *Rattus norvegicus *GenBank: AAL76339.1, *Mus musculus *UniProt: P51480-1, *Felis catus *GenBank: BAA33540.1, *Pan troglodytes *GenBank: XP_520513.1, *Homo sapiens *GenBank: NP_478104.1, CMT28 GenBank: FJ542308.1, NCF GenBank: FJ542309.1] and for p14^ARF ^[*Mesocricetus auratus *GenBank: AAN75824.1, *Rattus norvegicus *GenBank: AAL76336.1, *Mus sp*. GenBank: AAB35770.1, *Homo sapiens *GenBank: NP_478102.1].

In the flat-coated retrievers allele distribution between cases and controls was compared using a Monte Carlo simulation of the chi-square test and grouping together columns with expected values lower than 5, as implemented in the T2 test of the program Clump [62]; the level of significance was determined by performing 10000 simulations.

## Competing interests

The authors declare that they have no competing interests.

## Authors' contributions

JAH performed the LOH study, the sequencing of tumour samples, the DNA extraction and sequencing of blood samples, the alignments and bioinformatics analyses and wrote the manuscript. BM established the primary cell cultures, carried out the cytogenetic studies and made the chromosome paints. CQ performed the BAC mapping. PCMO flow-sorted the chromosomes. TH collected the biological samples and maintained the information database associated with it. SH performed the pathological diagnosis of the tumours and other biopsy samples. MAFS participated in the design of the study and in the interpretation of the results. JMD participated in the design of the project and in the collection of samples. DRS conceived and directed the project, interpreted the cytogenetic and BAC mapping results, analysed the sequencing data and participated in writing the manuscript. All authors read and approved the final manuscript.

## Supplementary Material

Additional file 1 **Distribution of *CDKN2B *exon 1 alleles in various dog breeds**. Cells show number of individuals for the corresponding genotypes and alleles. In the heading, genotypes and alleles are described in a simplified way (e.g., allele g. [109GGC[2];124GGGGACGGCGGC[3] appears as [2][3], while the heterozygous combination of g. [109GGC[2];124GGGGACGGCGGC[3] and g. [109GGC[5];124GGGGACGGCGGC[3] is shown as [2][3][5][3]. In the flat-coated retrievers, HS denotes histiocytic sarcoma). Click here for file

## References

[B1] Withrow SJ, Powers BE, Straw RC, Wilkins RM (1991). Comparative aspects of osteosarcoma. Dog versus man. Clin Orthop Relat Res.

[B2] Vail DM, MacEwen EG (2000). Spontaneously occurring tumors of companion animals as models for human cancer. Cancer Invest.

[B3] Hendrick MJ, Mahaffey EA, Moore FM, Vos JH, Walder EJ (1998). Histological classification of mesenchymal tumors of skin and soft tissues of domestic animals.

[B4] Sargan DR, Milne BS, Aguirre Hernandez J, O'Brien PC, Ferguson-Smith MA, Hoather T, Dobson JM (2005). Chromosome rearrangements in canine fibrosarcomas. J Hered.

[B5] Guyon R, Lorentzen TD, Hitte C, Kim L, Cadieu E, Parker HG, Quignon P, Lowe JK, Renier C, Gelfenbeyn B, Vignaux F, DeFrance HB, Gloux S, Mahairas GG, Andre C, Galibert F, Ostrander EA (2003). A 1-Mb resolution radiation hybrid map of the canine genome. Proc Natl Acad Sci USA.

[B6] Gil J, Peters G (2006). Regulation of the INK4b-ARF-INK4a tumour suppressor locus: all for one or one for all. Nat Rev Mol Cell Biol.

[B7] Ensembl. http://www.ensembl.org.

[B8] Lindblad-Toh K, Wade CM, Mikkelsen TS, Karlsson EK, Jaffe DB, Kamal M, Clamp M, Chang JL, Kulbokas EJ, Zody MC (2005). Genome sequence, comparative analysis and haplotype structure of the domestic dog. Nature.

[B9] Bignell GR, Santarius T, Pole JC, Butler AP, Perry J, Pleasance E, Greenman C, Menzies A, Taylor S, Edkins S, Campbell P, Quail M, Plumb B, Matthews L, McLay K, Edwards PA, Rogers J, Wooster R, Futreal PA, Stratton MR (2007). Architectures of somatic genomic rearrangement in human cancer amplicons at sequence-level resolution. Genome Res.

[B10] Nobori T, Miura K, Wu DJ, Lois A, Takabayashi K, Carson DA (1994). Deletions of the cyclin-dependent kinase-4 inhibitor gene in multiple human cancers. Nature.

[B11] Sharpless NE (2005). INK4a/ARF: a multifunctional tumor suppressor locus. Mutat Res.

[B12] Ruas M, Peters G (1998). The p16INK4a/CDKN2A tumor suppressor and its relatives. Biochim Biophys Acta.

[B13] Reef S, Zalckvar E, Shifman O, Bialik S, Sabanay H, Oren M, Kimchi A (2006). A short mitochondrial form of p19ARF induces autophagy and caspase-independent cell death. Mol Cell.

[B14] Reef S, Shifman O, Oren M, Kimchi A (2007). The autophagic inducer smARF interacts with and is stabilized by the mitochondrial p32 protein. Oncogene.

[B15] Ueda Y, Koya T, Yoneda-Kato N, Kato JY (2008). Small mitochondrial ARF (smARF) is located in both the nucleus and cytoplasm, induces cell death, and activates p53 in mouse fibroblasts. FEBS Lett.

[B16] Robertson KD, Jones PA (1999). Tissue-specific alternative splicing in the human INK4a/ARF cell cycle regulatory locus. Oncogene.

[B17] Lin YC, Diccianni MB, Kim Y, Lin HH, Lee CH, Lin RJ, Joo SH, Li J, Chuang TJ, Yang AS, Kuo HH, Tsai MD, Yu AL (2007). Human p16gamma, a novel transcriptional variant of p16(INK4A), coexpresses with p16(INK4A) in cancer cells and inhibits cell-cycle progression. Oncogene.

[B18] Krimpenfort P, Quon KC, Mooi WJ, Loonstra A, Berns A (2001). Loss of p16Ink4a confers susceptibility to metastatic melanoma in mice. Nature.

[B19] Sharpless NE, Bardeesy N, Lee KH, Carrasco D, Castrillon DH, Aguirre AJ, Wu EA, Horner JW, DePinho RA (2001). Loss of p16Ink4a with retention of p19Arf predisposes mice to tumorigenesis. Nature.

[B20] Kamijo T, Zindy F, Roussel MF, Quelle DE, Downing JR, Ashmun RA, Grosveld G, Sherr CJ (1997). Tumor suppression at the mouse INK4a locus mediated by the alternative reading frame product p19ARF. Cell.

[B21] Sharpless NE, Ramsey MR, Balasubramanian P, Castrillon DH, DePinho RA (2004). The differential impact of p16(INK4a) or p19(ARF) deficiency on cell growth and tumorigenesis. Oncogene.

[B22] Liu Q, Neuhausen S, McClure M, Frye C, Weaver-Feldhaus J, Gruis NA, Eddington K, Allalunis-Turner MJ, Skolnick MH, Fujimura FK (1995). CDKN2 (MTS1) tumor suppressor gene mutations in human tumor cell lines. Oncogene.

[B23] Lohmann DR (1999). RB1 gene mutations in retinoblastoma. Hum Mutat.

[B24] Drexler HG (1998). Review of alterations of the cyclin-dependent kinase inhibitor INK4 family genes p15, p16, p18 and p19 in human leukemia-lymphoma cells. Leukemia.

[B25] Merlo A, Herman JG, Mao L, Lee DJ, Gabrielson E, Burger PC, Baylin SB, Sidransky D (1995). 5' CpG island methylation is associated with transcriptional silencing of the tumour suppressor p16/CDKN2/MTS1 in human cancers. Nat Med.

[B26] Rocco JW, Sidransky D (2001). p16(MTS-1/CDKN2/INK4a) in cancer progression. Exp Cell Res.

[B27] Orlow I, Drobnjak M, Zhang ZF, Lewis J, Woodruff JM, Brennan MF, Cordon-Cardo C (1999). Alterations of INK4A and INK4B genes in adult soft tissue sarcomas: effect on survival. J Natl Cancer Inst.

[B28] Koenig A, Bianco SR, Fosmire S, Wojcieszyn J, Modiano JF (2002). Expression and significance of p53, rb, p21/waf-1, p16/ink-4a, and PTEN tumor suppressors in canine melanoma. Vet Pathol.

[B29] Levine RA, Fleischli MA (2000). Inactivation of p53 and retinoblastoma family pathways in canine osteosarcoma cell lines. Vet Pathol.

[B30] Fosmire SP, Thomas R, Jubala CM, Wojcieszyn JW, Valli VE, Getzy DM, Smith TL, Gardner LA, Ritt MG, Bell JS, Freeman KP, Greenfield BE, Lana SE, Kisseberth WC, Helfand SC, Cutter GR, Breen M, Modiano JF (2007). Inactivation of the p16 cyclin-dependent kinase inhibitor in high-grade canine non-Hodgkin's T-cell lymphoma. Vet Pathol.

[B31] DeInnocentes P, Agarwal P, Bird RC (2009). Phenotype-rescue of cyclin-dependent kinase inhibitor p16/INK4A defects in a spontaneous canine cell model of breast cancer. J Cell Biochem.

[B32] Korgaonkar C, Zhao L, Modestou M, Quelle DE (2002). ARF function does not require p53 stabilization or Mdm2 relocalization. Mol Cell Biol.

[B33] Llanos S, Clark PA, Rowe J, Peters G (2001). Stabilization of p53 by p14ARF without relocation of MDM2 to the nucleolus. Nat Cell Biol.

[B34] Kim SH, Mitchell M, Fujii H, Llanos S, Peters G (2003). Absence of p16INK4a and truncation of ARF tumor suppressors in chickens. Proc Natl Acad Sci USA.

[B35] Chen YJ, Chang JG, Shih LS, Chen PH, Endo M, Whang-Peng J, Chen YM (1997). Frequent detection of aberrant RNA transcripts of the CDKN2 gene in human gastric adenocarcinoma. Int J Cancer.

[B36] Cho JW, Jeong YW, Han SW, Park JB, Jang BC, Baek WK, Kwon TK, Park JW, Kim SP, Suh MH, Suh SI (2003). Aberrant p16INK4A RNA transcripts expressed in hepatocellular carcinoma cell lines regulate pRb phosphorylation by binding with CDK4, resulting in delayed cell cycle progression. Liver Int.

[B37] Baumgartner R, Fernandez-Catalan C, Winoto A, Huber R, Engh RA, Holak TA (1998). Structure of human cyclin-dependent kinase inhibitor p19INK4d: comparison to known ankyrin-repeat-containing structures and implications for the dysfunction of tumor suppressor p16INK4a. Structure.

[B38] Li J, Byeon IJ, Ericson K, Poi MJ, O'Maille P, Selby T, Tsai MD (1999). Tumor suppressor INK4: determination of the solution structure of p18INK4C and demonstration of the functional significance of loops in p18INK4C and p16INK4A. Biochemistry.

[B39] Li J, Mahajan A, Tsai MD (2006). Ankyrin repeat: a unique motif mediating protein-protein interactions. Biochemistry.

[B40] Lohi H, Young EJ, Fitzmaurice SN, Rusbridge C, Chan EM, Vervoort M, Turnbull J, Zhao XC, Ianzano L, Paterson AD, Sutter NB, Ostrander EA, Andre C, Shelton GD, Ackerley CA, Scherer SW, Minassian BA (2005). Expanded repeat in canine epilepsy. Science.

[B41] Fondon JW, Garner HR (2004). Molecular origins of rapid and continuous morphological evolution. Proc Natl Acad Sci USA.

[B42] Gilley J, Fried M (2001). One INK4 gene and no ARF at the Fugu equivalent of the human INK4A/ARF/INK4B tumour suppressor locus. Oncogene.

[B43] Krimpenfort P, Ijpenberg A, Song JY, Valk M van der, Nawijn M, Zevenhoven J, Berns A (2007). p15Ink4b is a critical tumour suppressor in the absence of p16Ink4a. Nature.

[B44] Byeon IJ, Li J, Ericson K, Selby TL, Tevelev A, Kim HJ, O'Maille P, Tsai MD (1998). Tumor suppressor p16INK4A: determination of solution structure and analyses of its interaction with cyclin-dependent kinase 4. Mol Cell.

[B45] Russo AA, Tong L, Lee JO, Jeffrey PD, Pavletich NP (1998). Structural basis for inhibition of the cyclin-dependent kinase Cdk6 by the tumour suppressor p16INK4a. Nature.

[B46] Li J, Poi MJ, Qin D, Selby TL, Byeon IJ, Tsai MD (2000). Tumor suppressor INK4: quantitative structure-function analyses of p18INK4C as an inhibitor of cyclin-dependent kinase 4. Biochemistry.

[B47] Hannon GJ, Beach D (1994). p15INK4B is a potential effector of TGF-beta-induced cell cycle arrest. Nature.

[B48] Sandhu C, Garbe J, Bhattacharya N, Daksis J, Pan CH, Yaswen P, Koh J, Slingerland JM, Stampfer MR (1997). Transforming growth factor beta stabilizes p15INK4B protein, increases p15INK4B-cdk4 complexes, and inhibits cyclin D1-cdk4 association in human mammary epithelial cells. Mol Cell Biol.

[B49] Reynisdottir I, Polyak K, Iavarone A, Massague J (1995). Kip/Cip and Ink4 Cdk inhibitors cooperate to induce cell cycle arrest in response to TGF-beta. Genes Dev.

[B50] Li JM, Nichols MA, Chandrasekharan S, Xiong Y, Wang XF (1995). Transforming growth factor beta activates the promoter of cyclin-dependent kinase inhibitor p15INK4B through an Sp1 consensus site. J Biol Chem.

[B51] Milne BS, Hoather T, O'Brien PC, Yang F, Ferguson-Smith MA, Dobson J, Sargan D (2004). Karyotype of canine soft tissue sarcomas: a multi-colour, multi-species approach to canine chromosome painting. Chromosome Res.

[B52] Li R, Mignot E, Faraco J, Kadotani H, Cantanese J, Zhao B, Lin X, Hinton L, Ostrander EA, Patterson DF, de Jong PJ (1999). Construction and characterization of an eightfold redundant dog genomic bacterial artificial chromosome library. Genomics.

[B53] Yang F, O'Brien PC, Milne BS, Graphodatsky AS, Solanky N, Trifonov V, Rens W, Sargan D, Ferguson-Smith MA (1999). A complete comparative chromosome map for the dog, red fox, and human and its integration with canine genetic maps. Genomics.

[B54] Benson G (1999). Tandem repeats finder: a program to analyze DNA sequences. Nucleic Acids Res.

[B55] Rozen S, Skaletsky J, Krawetz S, Misener S (2000). Primer3 on the WWW for general users and for biologist programmers. Bioinformatics Methods and Protocols: Methods in Molecular Biology.

[B56] Primer3 (v. 0.4.0). http://frodo.wi.mit.edu/primer3/.

[B57] HGVS, nomenclature for the description of sequence variations. http://www.hgvs.org/mutnomen/.

[B58] den Dunnen JT, Antonarakis SE (2000). Mutation nomenclature extensions and suggestions to describe complex mutations: a discussion. Hum Mutat.

[B59] Larkin MA, Blackshields G, Brown NP, Chenna R, McGettigan PA, McWilliam H, Valentin F, Wallace IM, Wilm A, Lopez R, Thompson JD, Gibson TJ, Higgins DG (2007). Clustal W and Clustal X version 2.0. Bioinformatics.

[B60] EMBL-EBI, ClustalW2. http://www.ebi.ac.uk/Tools/clustalw2/.

[B61] Clamp M, Cuff J, Searle SM, Barton GJ (2004). The Jalview Java alignment editor. Bioinformatics.

[B62] Sham PC, Curtis D (1995). Monte Carlo tests for associations between disease and alleles at highly polymorphic loci. Ann Hum Genet.

